# Quality of antibiotic prescribing to children through the coronavirus disease 2019 (COVID-19) pandemic

**DOI:** 10.1017/ash.2022.235

**Published:** 2022-06-15

**Authors:** Bethany A. Wattles, Kahir S. Jawad, Yana F. Feygin, J. Drew Stahl, Navjyot K. Vidwan, Michelle D. Stevenson, Maiying Kong, Michael J. Smith

**Affiliations:** 1 Department of Pediatrics, University of Louisville School of Medicine, Louisville, Kentucky; 2 Norton Children’s Research Institute Affiliated with University of Louisville School of Medicine, Louisville, Kentucky; 3 Department of Pharmacy, Norton Children’s Hospital, Louisville, Kentucky; 4 Norton Children’s and University of Louisville School of Medicine, Department of Pediatrics, Louisville, Kentucky; 5 Department of Bioinformatics and Biostatistics, University of Louisville School for Public Health and Information Sciences, Louisville, Kentucky; 6 Department of Pediatrics and Duke Center for Antimicrobial Stewardship and Infection Prevention, Duke University Medical Center, Durham, North Carolina

## Abstract

**Objective:**

To describe pediatric outpatient visits and antibiotic prescribing during the coronavirus disease 2019 (COVID-19) pandemic.

**Design:**

An observational, retrospective control study from January 2019 to October 2021.

**Setting:**

Outpatient clinics, including 27 family medicine clinics, 27 pediatric clinics, and 26 urgent or prompt care clinics.

**Patients:**

Children aged 0–19 years receiving care in an outpatient setting.

**Methods:**

Data were extracted from the electronic health record. The COVID-19 era was defined as April 1, 2020, to October 31, 2021. Virtual visits were identified by coded encounter or visit type variables. Visit diagnoses were assigned using a 3-tier classification system based on appropriateness of antibiotic prescribing and a subanalysis of respiratory visits was performed to compare changes in the COVID-19 era compared to baseline.

**Results:**

Through October 2021, we detected an overall sustained reduction of 18.2% in antibiotic prescribing to children. Disproportionate changes occurred in the percentages of antibiotic visits in respiratory visits for children by age, race or ethnicity, practice setting, and prescriber type. Virtual visits were minimal during the study period but did not result in higher rates of antibiotic visits or in-person follow-up visits.

**Conclusions:**

These findings suggest that reductions in antibiotic prescribing have been sustained despite increases in outpatient visits. However, additional studies are warranted to better understand disproportionate rates of antibiotic visits.

The coronavirus disease 2019 (COVID-19) pandemic and resultant mitigation strategies, social distancing, and mask mandates, have had a substantial impact on the epidemiology of other pediatric infections.^1–3^ At the same time, primary-care delivery transformed with the rapid increase in use of telemedicine.^4^ More recently, children returned to in-person school for the fall semester of 2021 during a surge in COVID-19 cases due to the severe acute respiratory coronavirus virus 2 (SARS-CoV-2) δ (delta) variant and an atypical summer–fall peak of respiratory syncytial virus (RSV).^5,6^ Antibiotic use is an important contributor to the development of antimicrobial resistance, and ∼30% of outpatient antibiotic prescribing is unnecessary.^7,8^ Understanding changes to antibiotic prescribing due to nontraditional viral respiratory patterns and novel care-delivery strategies is valuable for understanding and implementing outpatient antibiotic stewardship efforts.

In many outpatient settings serving children, the COVID-19 pandemic resulted in rapid implementation of telehealth visits.^4^ Virtual encounters, while offering numerous benefits, also have the potential to impact the quality of antibiotic prescribing, especially for conditions that benefit from physical exam and laboratory testing.^9^ Early studies showed significant reductions in antibiotic prescribing associated with the COVID-19 pandemic in the United States,^10–12^ and a recent study found sustained reductions in antibiotic prescribing in pediatric primary-care offices through June 2021, primarily driven by reductions in respiratory encounters.^13^ However, less is known about alternative outpatient settings, the influence of more recent changes in viral respiratory patterns (RSV and COVID-19 variants), return to in-person school, variations by patient or visit characteristics, and appropriateness of antibiotic prescribing. The primary objective of this study was to describe changes in antibiotic prescribing during the COVID-19 pandemic across a range of outpatient site and provider types. Secondary objectives were to evaluate changes in visit types, including (1) visits where antibiotics are sometimes, always, or never appropriate; (2) respiratory visits, the most common indications for outpatient antibiotic prescribing in children; and (3) telehealth visits and associated in-person follow-up visits.

## Methods

### Study design and setting

This retrospective control study utilized electronic health record (EHR) data from pediatric outpatient visits at Norton Healthcare, a large healthcare system serving the greater area of Louisville, Kentucky, and southern Indiana. Outpatient visit data were included for children aged 0–19 years from 27 family medicine or primary-care clinics, 27 pediatric clinics, and 26 urgent-care or retail clinics. In March 2020, 3 academic pediatric clinics were added to Norton Healthcare; in addition to expanding pediatric outpatient care, these are the only practice sites that include medical trainees. No formal outpatient antibiotic stewardship programs or interventions were conducted at Norton Healthcare during the study period. Visits were excluded if the provider type of record did not have authority to prescribe antibiotics (eg, nurse, medical assistant, registered dietician) or for encounter types not typically associated with medication prescribing (eg, imaging, injection visit, laboratory visit, social work). Outpatient visits were defined as virtual based on encounter and visit types, as coded in EHR variables, and we did not include telephone encounters. Virtual visits were counted as having a follow-up visit if any in-person visit type (eg, hospital, office visit, imaging, laboratory visit) for the same patient occurred within 48 hours.

A state of emergency was declared in Kentucky on March 6, 2020, in response to the COVID-19 pandemic.^14^ Beginning March 16–20, 2020, schools and daycare facilities were closed, and social distancing recommendations were implemented.^14^ In January 2021, schools were permitted to reopen for in-person learning, and legislation required in-person classes to resume by March 29, 2021.^14^ We defined a “baseline” (ie, prepandemic) period of January 1, 2019, to March 31, 2020. The “COVID-19 era” included April 1, 2020, to October 31, 2021. To calculate an absolute reduction in visits and antibiotic prescribing, we also compared corresponding periods of April–October in 2019, 2020, and 2021.

### Antibiotic prescribing and diagnoses

All included outpatient visits were queried for associated antibiotic prescribing through the EHR. The primary outcome of this study was percentage of antibiotic visits, defined as any visit with an associated oral antibiotic or select intramuscular antibiotic agents commonly used for pediatric infections (Supplementary Table A). Additional secondary outcomes of interest included changes in proportion of visit types, assessed by antibiotic appropriateness, all respiratory visits, and virtual visits. Visits were further sorted by diagnostic categories using *International Classification of Diseases, Tenth Edition* (ICD-10) codes. Visit appropriateness for antibiotic prescribing was defined using a previously described, mutually exclusive, ICD-10 classification scheme^16^ in which a diagnosis is classified as “always” appropriate for an antibiotic (eg, bacterial pneumonia, streptococcal pharyngitis), “sometimes” appropriate for an antibiotic (eg, acute sinusitis, acute otitis media), or “never” appropriate for an antibiotic (eg, acute upper respiratory infection, acute bronchitis). To identify a single diagnosis for each visit, modified clinical classification software categories^15^ were prioritized using a similar tiered classification scheme. Classification was hierarchical such that if any of the listed medical diagnoses should always be treated with an antibiotic, the “always” diagnosis was prioritized over diagnoses for which antibiotics are sometimes or never indicated, respectively. For indications for which antibiotics are never indicated, we prioritized reporting of the following infectious categories of interest: acute upper respiratory infection, acute bronchitis (including bronchiolitis), influenza, and COVID-19. If >1 diagnosis was identified per tiered category, the first reported diagnosis field was selected. COVID-19 visits were defined using ICD-10 codes for diagnosis, exposure, screening, or personal history (ie, U071, Z20822, Z1152, Z8616, respectively). Respiratory visits of interest were identified using modified clinical classification software categories^15^ and included bronchitis, COVID-19, influenza, otitis media, pharyngitis, pneumonia, sinusitis, tonsillitis, and other upper respiratory infections (eg, laryngitis). A full list of diagnosis classifications is available in Supplementary Table B.

### Data collection

This study was deemed exempt from approval by the Institutional Review Board of the University of Louisville and the Norton Healthcare Research Office. Patient and visit characteristics, antibiotic prescriptions, and ICD-10 codes were extracted from the EHR database. Race and ethnicity were assigned using the primary variable collected in the EHR and were recategorized as White, Black, Hispanic, other, and unknown.

### Data analysis

Descriptive statistics were used to summarize outpatient visits and antibiotic prescribing stratified by period (ie, baseline vs COVID-19 era), month, visit diagnoses of interest, and virtual visits with in-person follow-up within 48 hours. Reductions in the percentages of antibiotic visits in the COVID-19 era for respiratory visits were calculated as relative reductions compared to the baseline era.

We applied multivariable logistic regression models to identify which characteristics were associated with antibiotic prescribing in respiratory visits during the study period. The following factors were included in the analysis: period (baseline vs COVID-19 era); visit type (in-person vs virtual); sex, age group (0–2 years, 3–9 years, or 10–19 years); race or ethnicity (White, Black, Hispanic, or other); insurance (Medicaid vs private); department (family medicine, pediatrics, retail clinics, or urgent care); and provider type (physician, nurse practitioner, or trainee). Odds ratios (ORs) and their 95% confidence intervals (95% CIs) were calculated. All statistical analyses were carried out using SAS version 9.4 software (SAS Institute, Cary, NC).

## Results

During the study period, 984,244 outpatient visits were included among 198,315 unique patients. From April to October 2019, there were 197,253 outpatient visits, compared to 158,497 visits in the same period of 2020 and 244,189 visits in 2021. In the same monthly periods (April–October), there were 40,140 visits during which antibiotics were prescribed in 2019, compared to 16,396 antibiotic visits in the same period of 2020. These data represent a 59.2% reduction in antibiotic prescribing from 2019 to 2020. During the follow-up period of April–October 2021, there were 32,820 antibiotic visits, an 18.2% reduction in antibiotic prescribing compared to the baseline. The percentage reduction in antibiotic prescriptions during respiratory visits during only the corresponding April–October periods were larger: 71.2% in 2020 and 23.2% in 2021.

The proportion of visits by children who were younger, non-White, and insured by Medicaid increased during the COVID-19 era, as did visits to pediatric clinics (Table 1). The percentages of antibiotic visits decreased for all patient characteristics in the COVID-19 era. Frequency of visits over time by appropriateness for antibiotics (always, sometimes, or never), and rates of antibiotic prescriptions per 1,000 visits are summarized in Figure 1a. This figure shows sharp declines in response to initial stay-at-home orders (beginning March 2020) and subsequent increases in visits beginning around March 2021.

**Table 1.** Characteristics of Outpatient Visits, January 2019-October 2021

Characteristic	Overall	Baseline, No. (%)		COVID-19 Era, No. (%)	
	No. (%)	Visits	Antibiotic Visits^a^	Visits	Antibiotic Visits^a^
Overall	984,244	455,264	101,721 (**22.3**)	528,980	62,157 (**11.8**)
**Visit type**					
In-person	975,592 (**99.1**)	454,827 (**99.9**)	101,627 (**22.3**)	520,765 (**98.4**)	61,382 (**11.8**)
Telehealth	8,652 (**0.9**)	437 (**0.1**)	94 (**21.5**)	8,215 (**1.6**)	775 (**9.4**)
**Sex**					
Female	492,762 (**50.1**)	227,135 (**49.9**)	52,024 (**22.9**)	265,627 (**50.2**)	33,014 (**12.4**)
Male	491,445 (**49.9**)	228,120 (**50.1**)	49,693 (**21.8**)	263,325 (**49.8**)	29,142 (**11.1**)
**Age group**					
0–2 y	394,002 (**40.0**)	176,218 (**38.7**)	32,208 (**18.3**)	217,784 (**41.2**)	22,609 (**10.4**)
3–9 y	295,446 (**30.0**)	145,596 (**32.0**)	41,394 (**28.4**)	149,850 (**28.3**)	20,125 (**13.4**)
10–19 y	294,796 (**30.0**)	133,450 (**29.3**)	28,119 (**21.1**)	161,346 (**30.5**)	19,423 (**12.0**)
**Race/ethnicity**					
White	641,363 (**65.2**)	309,705 (**68.0**)	73,973 (**23.9**)	331,658 (**62.7**)	43,005 (**13.0**)
Black	173,943 (**17.7**)	71,338 (**15.7**)	13,421 (**18.8**)	102,605 (**19.4**)	9,888 (**9.6**)
Hispanic	66,890 (**6.8**)	28,803 (**6.3**)	6,379 (**21.1**)	38,087 (**7.2**)	4,233 (**11.1**)
Other	41,739 (**4.2**)	19,356 (**4.3**)	3,634 (**18.8**)	22,383 (**4.2**)	1,872 (**8.4**)
Unknown	60,309 (**6.1**)	26,062 (**5.7**)	4,314 (**16.6**)	34,247 (**6.5**)	3,159 (**9.2**)
**Insurance**					
Medicaid	529,039 (**53.8**)	234,615 (**51.5**)	49,232 (**21.0**)	294,424 (**55.7**)	32,947 (**11.2**)
Private	453,683 (**46.1**)	219,899 (**48.3**)	52,393 (**23.8**)	233,784 (**44.2**)	29,139 (**12.5**)
Other	1,522 (**0.2**)	750 (**0.2**)	96 (**12.8**)	772 (**0.1**)	71 (**9.2**)
**Department**					
Family medicine/Primary care	60,507 (**6.2**)	30,688 (**6.7**)	4,390 (**14.3**)	29,819 (**5.6**)	2,288 (**7.7**)
Pediatrics	756,491 (**76.9**)	339,857 (**74.7**)	66,054 (**19.4**)	416,634 (**78.8**)	39,490 (**9.5**)
Urgent care	155,906 (15.8)	79,083 (17.4)	28,686 (36.3)	76,823 (14.5)	18,890 (24.6)
Retail clinics	11,340 (1.15)	5,636 (1.2)	2,591 (46.0)	5,704 (1.1)	1,489 (26.1)
**Provider type**					
Nurse practitioner	215,918 (**21.9**)	95,585 (**21.0**)	28,988 (**30.3**)	120,333 (**22.7**)	21,462 (**17.8**)
Physician	740,478 (**75.2**)	354,218 (**77.8**)	72,001 (**20.3**)	386,260 (**73.0**)	39,389 (**10.2**)
Trainee	20,966 (**2.1**)	1,729 (**0.4**)	380 (**22.0**)	19,237 (**3.6**)	1,153 (**6.0**)
Physician assistant	6,882 (**0.7**)	3,732 (**0.8**)	352 (**9.4**)	3,150 (**0.6**)	153 (**4.9**)

Note. The baseline period was January 1, 2019, to March 31, 2020, and the COVID-19 era was defined as April 1, 2020, to October 31, 2021.

a Antibiotic visit percentages calculated as percentage of visits where an antibiotic was prescribed (row percentages).

**Fig. 1a.**
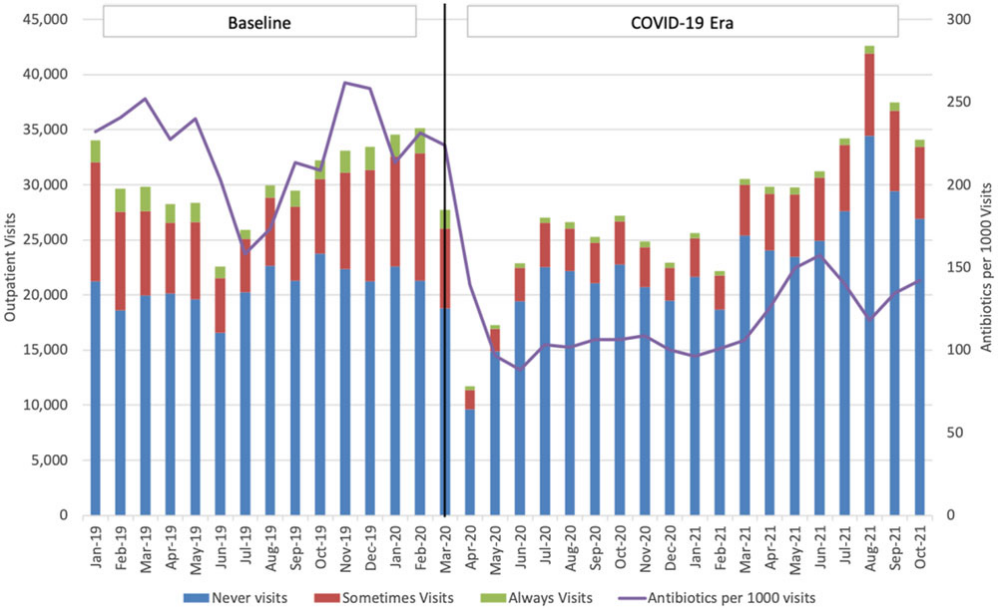
Trends in outpatient visits and antibiotic prescribing per 1,000 visits. Outpatient visits summarized by appropriateness of antibiotics for diagnoses (never, sometimes, always). Vertical line represents start of the COVID-19 era.

Relative changes in the proportion of visits that were “always,” “sometimes,” or “never” appropriate for antibiotics are presented in Figure 1b. “Sometimes” visits gradually increased from 10% to 20% of all visits in the COVID-19 era, but this did not reach the peak seasonal highs of 30% observed prior to the pandemic. “Always” visits remained reduced throughout the COVID-19 era. Antibiotic prescribing for “sometimes” visits decreased from 51.7% to 48.4% from baseline to the COVID-19 era, whereas prescribing for “never” visits decreased from 5.4% to 2.86%. Reductions over time were greater for acute upper respiratory infections, but nonrespiratory infections (eg, skin and soft-tissue infection and urinary tract infection) remained stable over the study period (Fig. 2).

**Fig. 1b.**
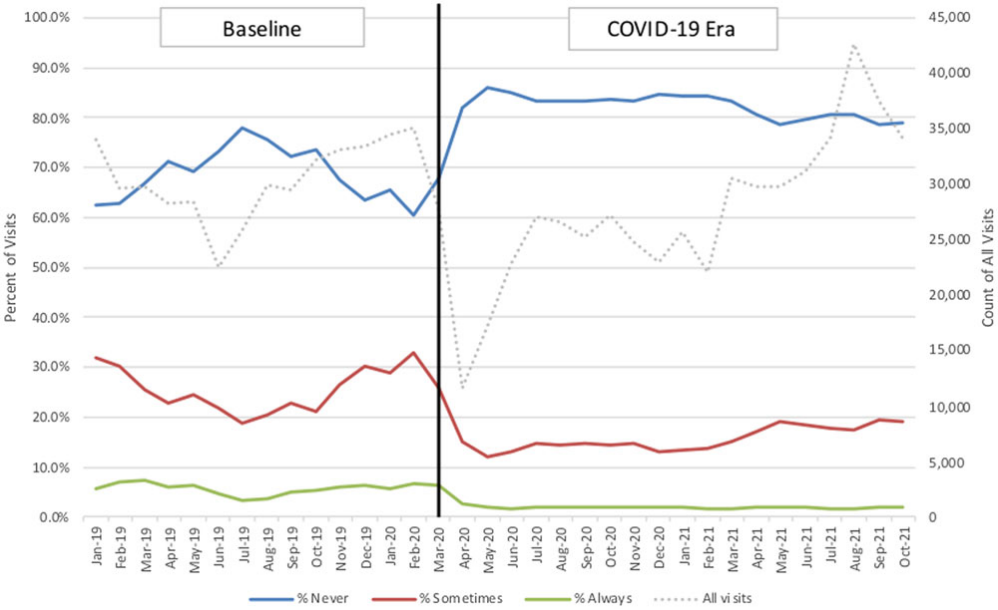
Changes in proportion of visits over time by antibiotic appropriateness. Top diagnoses for “always” visits include streptococcal pharyngitis, urinary tract infection, and bacterial pneumonia. Top diagnoses for “sometimes” visits include pharyngitis (not specified), acute otitis media, acute sinusitis, and skin and soft-tissue infection (SSTI).

**Fig. 2.**
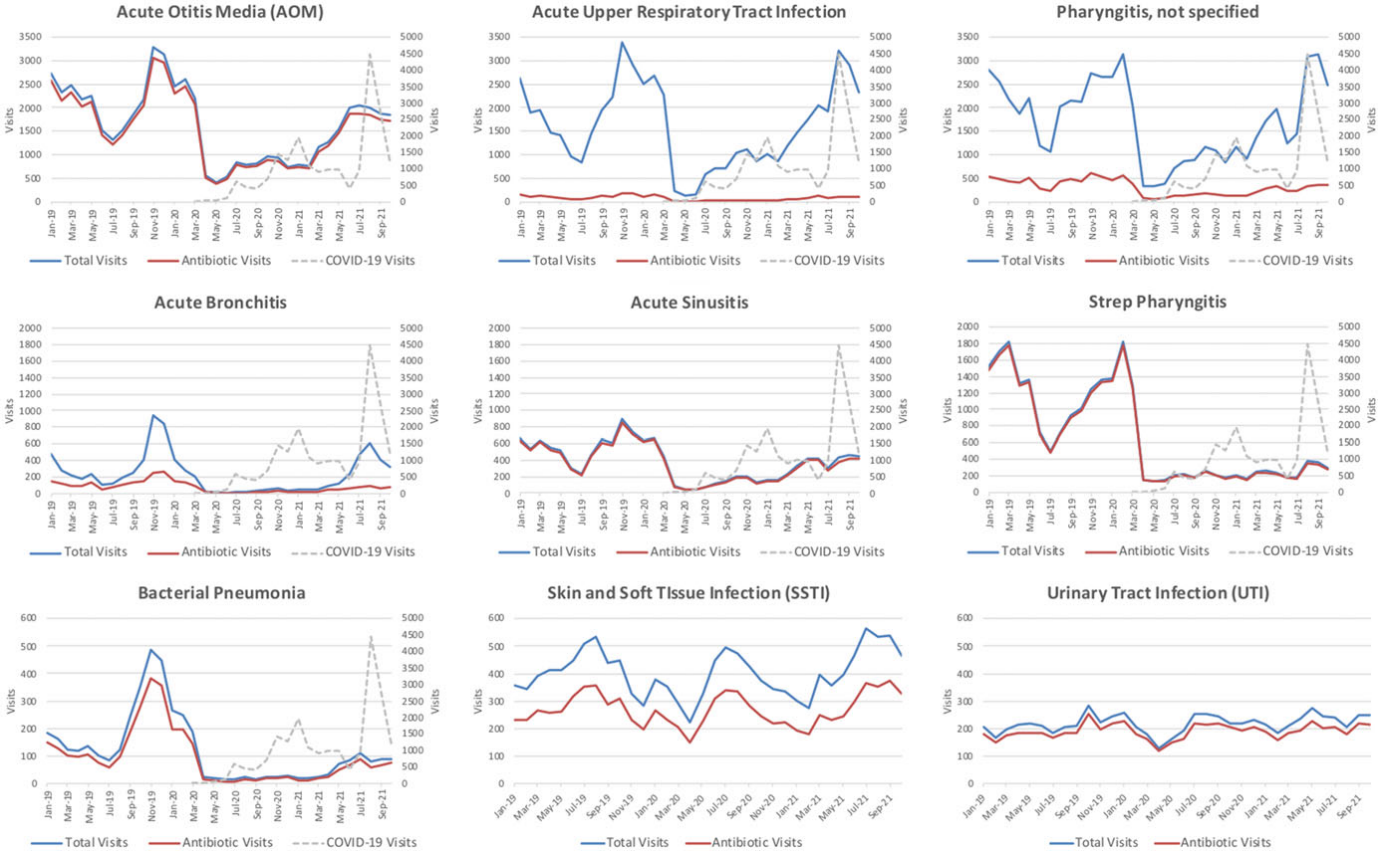
Changes in diagnoses of interest and associated antibiotic prescribing over time. Changes were present in respiratory diagnoses, while non-respiratory diagnoses of SSTI and UTI remained unchanged. Note: Y-axes match across rows but differ between rows. Visits with concern for COVID-19 (exposure, testing, or disease) are shown as trend lines for reference on respiratory diagnosis graphs.

Subanalyses were performed for pediatric respiratory visits. We detected a 29.4% relative reduction in percentage of antibiotic visits from baseline to the COVID-19 era (Table 2). Reductions in percentage of antibiotic visits were highest in the following groups: children aged 3–9 years (36.4%) and 10–19 years (42.7%), Black children (32.4%), privately insured children (31.7%), and children seen at urgent-care or retail clinics (39.6%). A multivariable regression model revealed that the odds of receiving an antibiotic prescription for a respiratory visit decreased ∼50% during the COVID-19 era versus baseline (OR, 0.548; CI, 0.539–0.557) and 15% in virtual visits versus in-person visits (OR, 0.855; CI, 0.755–0.969), adjusted for patient and visit characteristics (Table 3).

**Table 2.** Characteristics of Respiratory Outpatient Visits and Relative Reductions in Antibiotic visits, January 2019–October 2021

Characteristic	Overall No. (%)	Baseline, No. (%)		COVID-19 Era, No. (%)		Relative Reduction in Antibiotic Visits, %
		Respiratory Visits	Antibiotic Visits^a^	Respiratory Visits	Antibiotic Visits^a^	
Overall	272,932	161,115	80,874 (50.2)	111,817	39,632 (35.4)	29.4
**Visit type**						
In-person	271,658 (99.5)	160,962 (99.9)	80,815 (50.2)	110,696 (99.0)	39,296 (35.5)	29.3
Telehealth	1,274 (0.5)	153 (0.1)	59 (38.6)	1,121 (1.0)	336 (30.0)	22.3
**Sex**						
Female	136,851 (50.1)	80,629 (50.0)	39,827 (49.4)	56,222 (50.3)	19,484 (34.7)	29.8
Male	136,081 (49.9)	80,486 (50.0)	41,047 (51.0)	55,595 (49.7)	20,148 (36.2)	29.0
**Age group**						
0–2 y	92,965 (34.1)	53,486 (33.2)	27,264 (51.0)	39,479 (35.3)	18,138 (45.9)	10.0
3–9 y	99,247 (36.4)	63,053 (39.1)	34,077 (54.1)	36,194 (32.4)	12,433 (34.4)	36.4
10–19 y	80,720 (29.6)	44,576 (27.7)	19,533 (43.8)	36,144 (32.3)	9,061 (25.1)	42.7
**Race/ethnicity**						
White	189,718 (69.5)	114,764 (71.2)	59,203 (51.6)	74,954 (67.0)	27,598 (36.8)	28.7
Black	40,751 (14.9)	22,057 (13.7)	10,209 (46.3)	18,694 (16.7)	5,849 (31.3)	32.4
Hispanic	18,231 (6.7)	10,362 (6.4)	5,096 (49.2)	7,869 (7.0)	2,843 (36.1)	26.6
Other	10,150 (3.7)	6,313 (3.9)	2,930 (46.4)	3,837 (3.4)	1,210 (31.5)	32.1
Unknown	14,082 (5.2)	7,619 (4.7)	3,436 (45.1)	6,463 (5.8)	2,132 (33.0)	26.8
**Insurance**						
Medicaid	140,638 (51.5)	80,934 (50.2)	38,914 (48.1)	59,704 (53.4)	21,038 (35.2)	26.8
Private	132,083 (48.4)	80,076 (49.7)	41,907 (52.3)	52,007 (46.5)	18,581 (35.7)	31.7
**Department**						
Family medicine/primary care	10,233 (3.8)	6,782 (4.2)	3,057 (45.1)	3,451 (3.1)	1,106 (32.1)	28.8
Pediatrics	176,320 (64.6)	107,107 (66.5)	53,630 (50.1)	69,213 (61.9)	26,434 (38.2)	23.8
Urgent care	78,438 (28.7)	43,127 (26.8)	21,859 (50.7)	35,311 (31.6)	10,943 (31.0)	38.9
Retail clinics	7,947 (2.9)	4,102 (2.6)	2,330 (56.8)	3,845 (3.4)	1,150 (29.9)	47.4
**Provider type**						
Nurse practitioner	82,781 (30.3)	44,161 (27.4)	23,279 (52.7)	38,620 (34.5)	13,859 (35.9)	31.9
Physician	185,086 (67.8)	115,212 (71.5)	57,033 (49.5)	69,874 (62.5)	25,233 (36.1)	27.1
Trainee	3,640 (1.3)	775 (0.5)	278 (35.9)	2,865 (2.6)	454 (15.9)	55.7
Physician assistant	1,425 (0.5)	967 (0.6)	284 (29.4)	458 (0.4)	86 (18.8)	36.1

Note. The baseline period was January 1, 2019, to March 31, 2020, and the COVID-19 era was defined as April 1, 2020, to October 31, 2021.

a Antibiotic visit percentages calculated as percentage of respiratory visits where an antibiotic was prescribed (row percentages).

**Table 3.** Adjusted Odds Ratios for Antibiotic Prescribing in Respiratory Visits

Variable	Odds Ratio	95% Wald Confidence Limits		*P* Value
**Period**				
Baseline	Reference			
COVID-19 era	0.546	0.537	0.556	<.0001
**Visit type**				
In-person	Reference			
Virtual	0.846	0.747	0.958	.0083
**Sex**				
Female	Reference			
Male	1.052	1.035	1.069	<.0001
**Race or ethnicity**				
Black	Reference			
White	1.27	1.24	1.3	<.0001
Hispanic	1.169	1.126	1.213	<.0001
Other	0.97	0.926	1.016	.2023
**Insurance**				
Medicaid	Reference			
Private	1.125	1.106	1.144	<.0001
**Department**				
Family medicine/Primary care	Reference			
Pediatrics	1.209	1.157	1.263	<.0001
Urgent care	1.144	1.094	1.197	<.0001
Retail clinics	1.098	1.028	1.172	.0055
**Provider type**				
Physician	Reference			
Nurse practitioner	1.184	1.161	1.208	<.0001
Trainee	0.424	0.389	0.462	<.0001
**Age group**				
10–19 y	Reference			
0–2 y	1.852	1.812	1.893	<.0001
3–9 y	1.598	1.566	1.631	<.0001

### Virtual visits

There were 8,652 virtual visits during the study period: 437 (5.1%) were in the baseline period and 8,215 (95.0%) were in the COVID-19 era. Also, 715 (8.3%) of all virtual visits had in-person follow-up visits within 48 hours (Table 4). The rates of follow-up were higher for female patients (9.2%), for children aged 0–2 years (10.8%), for visits at urgent-care clinics (15.8%), and for visits with nurse practitioners (14.0%). Visits for viral infections (including acute bronchitis and acute upper-respiratory infections) had a higher follow-up rate (17%), as did visits for pharyngitis (including *Streptococcus* and non-*Streptococcus* diagnoses, 16.9%) and COVID-19 (20.6%). However, AOM had a follow-up rate of 6.1%, sinusitis had a follow-up rate of 7.1%, and other diagnoses had a follow-up rate of 7.1%.

**Table 4.** Virtual Visits and In-Person Follow-up Within 48 Hours

Variable	Virtual Visits, No. (%)	Virtual Visits with Follow-Up, No. (%)^a^
**Overall**	8,652	715 (8.3)
**Period**		
Baseline	437 (5.1)	44 (10.1)
COVID-19 era	8,215 (95.0)	671 (8.2)
**Sex**		
Female	4,244 (49.1)	389 (9.2)
Male	4,408 (51.0)	326 (7.4)
**Age group**		
0–2 y	1,387 (16.0)	150 (10.8)
3–9 y	3,100 (35.8)	248 (8.0)
10–19 y	4,165 (48.1)	317 (7.6)
**Race/ethnicity**		
White	6,519 (75.4)	521 (8.0)
Black	1,332 (15.4)	131 (9.8)
Hispanic	274 (3.2)	26 (9.5)
Other	222 (2.6)	27 (12.2)
Unknown	305 (3.5)	10 (3.3)
**Insurance**		
Medicaid	4,062 (47.0)	352 (8.7)
Private	4,587 (53.0)	363 (7.9)
Other	3 (0.0)	0 (0.0)
**Department**		
Family medicine/primary care	1,816 (0.2)	141 (7.8)
Pediatrics	4,272 (62.7)	170 (4.0)
Urgent care	2,493 (28.8)	400 (16.0)
Retail clinics	71 (0.8)	4 (5.6)
**Provider type**		
Nurse practitioner	3,397 (39.3)	474 (14.0)
Physician	5,188 (60.0)	241 (4.6)
Trainee	57 (0.7)	0 (0.0)
Physician assistant	10 (0.1)	0 (0.0)
**Diagnoses**		
Viral^b^	353 (4.1)	60 (17.0)
Pharyngitis^c^	337 (3.9)	57 (16.9)
Acute otitis media	66 (0.8)	4 (6.1)
Sinusitis	154 (1.8)	11 (7.1)
COVID-19	243 (2.8)	50 (20.6)
Other	7,499 (86.7)	533 (7.1)

Note. The baseline period was January 1, 2019, to March 31, 2020, and the COVID-19 era was defined as April 1, 2020, to October 31, 2021.

a Follow-up included all visit types (laboratory, imaging, office visits, hospitalizations, etc).

b Viral diagnoses include acute bronchitis and acute upper respiratory infection.

c Pharyngitis diagnoses include streptococcal pharyngitis and pharyngitis not specified.

## Discussion

Our study describes a 59.2% reduction in antibiotic prescribing to children during the height of social isolation due to COVID-19 in 2020 and an 18.2% reduction in antibiotic prescribing in the same seasonal period in 2021, despite an overall increase in the number of outpatient visits in 2021. We detected greater reductions in antibiotic prescribing for respiratory illnesses: 71.2% in 2020 and 23.2% in 2021. This partially sustained reduction in antibiotic prescriptions occurred in the setting of return to in-person learning and likely substantial increases in social gatherings outside school. A portion of this reduction in antibiotic prescribing could be explained by continued masking in schools and/or public settings. However, this period also included an atypical RSV season; local cases reached a higher peak in August 2021 than typically seen in winter months, suggesting effective transmission of respiratory pathogens in the community. Typical respiratory viruses (eg, RSV and influenza) have been shown to directly correlate with seasonal increases in antibiotic prescribing both before and during the COVID-19 pandemic.^17^

Our findings show dramatic changes in respiratory diagnoses over the study period but minimal changes to nonrespiratory diagnoses (Fig. 2). Similarly, these findings are consistent in our analyses of appropriateness of antibiotic prescribing based on visit diagnoses (“always,” “sometimes,” and “never” visits). Sharp declines in diagnoses and antibiotic visits in March–May 2020 have been previously explained.^10–13^ However, the changes detected in May–September 2021 are novel. For instance, initial declines in AOM diagnoses were likely because AOM is most frequently a consequence of viral upper-respiratory infection,^18^ and viral infections decreased due to social distancing and masking. AOM cases increased again in summer 2021when acute upper-respiratory infections peaked.

Previous studies showed similar declines in respiratory diagnoses and antibiotic prescribing during earlier stages of the COVID-19 pandemic.^10–13^ Evaluating national prescribing data, Chua et al^19^ reported a 55.6% reduction in antibiotic prescribing to children in April–December 2020 compared to the corresponding period in 2019. More recently, investigators described a 72.7% reduction in antibiotic prescriptions within a study period from January 2019 through June 2021 in pediatric primary-care practices associated with the Children’s Hospital of Philadelphia.^13^ Our study expands on the existing literature by including fall 2021 data, which included return to school and increases in circulating respiratory viruses as previously described. We also included nonpediatric primary-care settings such as family medicine and urgent care and retail practices.

Additionally, we identified disproportionate changes in antibiotic prescribing by patient characteristics, prescriber type, and practice settings. Earlier publications evaluating antibiotic prescribing during the pandemic have reported on monthly trends and relative changes in antibiotic prescribing beyond expected, using monthly matched time periods.^11,12^ However, in 2021, the RSV peak and SARS-CoV-2 δ (delta) variant produced unseasonably high rates of viral illness in summer–fall months. Therefore, we chose to report percent reductions in antibiotic prescribing in respiratory visits, to account for fluctuations in respiratory visits throughout the study period.

The COVID-19 pandemic has disproportionately affected adults and children by race and social status with regard to illness, use of telemedicine, and ability to practice social distancing.^20–23^ Additionally, prior to the COVID-19 pandemic, White children were prescribed antibiotics at higher rates than Black or non-White children.^24–26^ Although the reduction in antibiotic visits could represent reduced inappropriate prescribing, it could also potentially represent undertreatment and/or limitations in access to care. The racial discrepancy identified in our study may represent another example of how racism has caused widened health disparities through the COVID-19 pandemic^27^ and warrants further study because this factor could have greater implications for other areas of pediatric care.

Our study also showed greater decreases in antibiotic visits for older children aged ≥3 years, possibly due to virtual learning and masking or shifts in parental expectations for antibiotics in the setting of viral illness. For instance, caregivers could be more focused on obtaining testing for COVID-19 for return to school and less likely to expect antibiotics for a child’s respiratory illness. These findings suggest that there could be a favorable shift in culture and expectations surrounding outpatient visits for acute upper respiratory infections, representing a reduction in unnecessary antibiotic prescribing. Continued monitoring and reporting of antibiotic prescribing trends are warranted. We also saw disproportionate changes in practice settings, likely reflective of the addition of academic pediatric practices that joined the healthcare system in March 2020. Similarly, trainees are only involved in outpatient care in the newly added pediatric clinics and therefore had an increase in respiratory visits during the COVID-19 era.

Differences in relative reduction of antibiotic visits by practice setting and provider type could be attributable to several changes throughout the pandemic. For instance, reductions were greatest in urgent care and retail clinics which could be due to such settings being more frequently visited for COVID-19 tests as the primary focus of the visit. Physician assistants also experienced greater reductions in antibiotic visits compared to nurse practitioners and physicians; however, physician assistants are uncommon in pediatric primary care at our institution and results likely reflect the prescribing practices of a few providers.

Prior to the COVID-19 pandemic, studies evaluating antibiotic prescribing in virtual visits compared to in-person visits had conflicting and controversial results.^28–30^ Although virtual visits in our study sample were low, our findings suggest that antibiotic prescribing for respiratory diagnoses was less common in virtual visits (Table 3). Additionally, a subanalysis of virtual visits showed that rates of in-person follow-up were not high for common bacterial diagnoses of AOM and sinusitis (6.1% and 7.1% follow-up, respectively). Alternatively, we did see higher rates of in-person follow-up visits (16%–20%) for diagnoses that could benefit from laboratory testing, such as pharyngitis, COVID-19, and other respiratory viral infections. Given the low rates of virtual and follow-up visits, additional analyses of antibiotic prescribing in these visits were not performed.

This study had several limitations. Our study sample included pediatric visits to a single healthcare organization, and findings may not be generalizable to other organizations or geographic settings. We were unable to quantify any changes in pediatric visits due to patient relocation or care-seeking behaviors. We utilized ICD-10 codes to identify visit diagnoses, a common practice for database research in antimicrobial stewardship; however, accuracy between diagnostic billing and clinical diagnosis cannot be guaranteed. We utilized respiratory visits to compare changes in antibiotic prescribing and in the regression model, to standardize time periods for seasonal variations. We chose to include COVID-19 visits because COVID-19 may present with respiratory symptoms similar to other viral indications for which antibiotics are commonly prescribed inappropriately. However, inclusion produces lower rates of antibiotic visits in the COVID-19 era. We included medical practices that were added to our organization during the study period; however, sensitivity analyses revealed that the addition of these practices did not impact odds of an antibiotic visit. Finally, we included all visit types for our virtual follow-up visit analysis, which could have included routinely scheduled visits and, therefore, we may have overestimated the rate of in-person follow-up visits.

In conclusion, we report decreases in antibiotic prescribing during the COVID-19 pandemic, with disproportionate changes among patient groups by age, race or ethnicity, insurance status, and practice setting. Additional qualitative studies are needed to better understand changes in culture and expectations surrounding antibiotic prescribing during this unprecedented time. Tracking and reporting of antibiotic prescribing is a key component of outpatient stewardship initiatives. Efforts should be made for organizations to share such data with prescribing clinicians to continue to promote favorable trends in judicious antibiotic use.

## Supplementary material

For supplementary material accompanying this paper visit https://doi.org/10.1017/ash.2022.235. click here to view supplementary material

## References

[1] Hatoun J , Correa ET , Donahue SMA , Vernacchio L. Social distancing for COVID-19 and diagnoses of other infectious diseases in children. Pediatrics 2020;146(4):e2020006460. 32879032 10.1542/peds.2020-006460

[2] Schweiberger K , Patel SY , Mehrotra A , Ray KN. Trends in pediatric primary care visits during the coronavirus disease of 2019 pandemic. Acad Pediatr 2021;21:1426–1433. 33984496 10.1016/j.acap.2021.04.031 PMC8561008

[3] Haddadin Z , Schuster JE , Spieker AJ , et al. Acute respiratory illnesses in children in the SARS-CoV-2 pandemic: prospective multicenter study. Pediatrics 2021;148(2):e2021051462. 33986150 10.1542/peds.2021-051462 PMC8338906

[4] Fiks AG , Jenssen BP , Ray KN. A defining moment for pediatric primary care telehealth. JAMA Pediatr 2021;175:9–10. 32658256 10.1001/jamapediatrics.2020.1881

[5] Brown CM , Vostok J , Johnson H , et al. Outbreak of SARS-CoV-2 infections, including COVID-19 vaccine breakthrough infections, associated with large public gatherings—Barnstable County, Massachusetts, July 2021. Morb Mortal Wkly Rep 2021;70:1059–1062. 10.15585/mmwr.mm7031e2 PMC8367314 34351882

[6] Agha R , Avner JR. Delayed seasonal RSV surge observed during the COVID-19 pandemic. Pediatrics 2021;148(3):e2021052089. 34108234 10.1542/peds.2021-052089

[7] Antibiotic resistance threats in the United States, 2019. Centers for Disease Control and Prevention website. https://www.cdc.gov/drugresistance/pdf/threats-report/2019-ar-threats-report-508.pdf. Published 2019. Accessed May 26, 2022.

[8] Fleming-Dutra KE , Hersh AL , Shapiro DJ , et al. Prevalence of inappropriate antibiotic prescriptions among US ambulatory care visits, 2010–2011. JAMA 2016;315:1864–1873. 27139059 10.1001/jama.2016.4151

[9] Suzuki H , Marra AR , Hasegawa S , et al. Outpatient antibiotic prescribing for common infections via telemedicine versus face-to-face visits: systematic literature review and meta-analysis. Antimicrob Steward Healthc Epidemiol 2021;1:E24. 36168456 10.1017/ash.2021.179 PMC9495625

[10] Katz SE , Spencer H , Zhang M , Banerjee R. Impact of the COVID-19 pandemic on infectious diagnoses and antibiotic use in pediatric ambulatory practices. J Pediatric Infect Dis Soc 2021;10:62–64. 33064837 10.1093/jpids/piaa124 PMC7797736

[11] King LM , Lovegrove MC , Shehab N , et al. Trends in US outpatient antibiotic prescriptions during the coronavirus disease 2019 pandemic. Clin Infect Dis 2021;73:e652–e660. 33373435 10.1093/cid/ciaa1896 PMC7799289

[12] Buehrle DJ , Wagener MM , Nguyen MH , Clancy CJ. Trends in outpatient antibiotic prescriptions in the United States during the COVID-19 pandemic in 2020. JAMA Netw Open 2021;4(9):e2126114. 34550387 10.1001/jamanetworkopen.2021.26114 PMC8459187

[13] Dutcher L , Li Y , Lee G , Grundmeier R , Hamilton KW , Gerber JS , for the CDC Epicenters Program. COVID-19 and Antibiotic prescribing in pediatric primary care. Pediatrics 2022;149(2):e2021053079. 35102416 10.1542/peds.2021-053079 PMC9825803

[14] Kentucky’s response to COVID-19. Kentucky Governor website. https://governor.ky.gov/covid19. Accessed February 2, 2022.

[15] Clinical Classifications Software (CCS) for ICD-10-PCS (beta version). Agency for Healthcare Research Quality, Healthcare Cost and Utilization Project website. https://www.hcup-us.ahrq.gov/toolssoftware/ccs10/ccs10.jsp#overview. Published November 2019. Accessed January 26, 2022.

[16] Chua KP , Fischer MA , Linder JA. Appropriateness of outpatient antibiotic prescribing among privately insured US patients: ICD-10-CM–based cross-sectional study. BMJ 2019;364:k5092. 30651273 10.1136/bmj.k5092 PMC6334180

[17] Lepak AJ , Taylor LN , Stone CA , et al. Association of Changes in seasonal respiratory virus activity and ambulatory antibiotic prescriptions with the COVID-19 pandemic. JAMA Intern Med 2021;181:1399–1402. 34152385 10.1001/jamainternmed.2021.2621 PMC8218231

[18] Lieberthal AS , Carroll AE , Chonmaitree T , et al. The diagnosis and management of acute otitis media. Pediatrics 2013;131:e964–e999. 23439909 10.1542/peds.2012-3488

[19] Chua KP , Volerman A , Conti RM. Prescription drug dispensing to US children during the COVID-19 pandemic. Pediatrics 2021;148(2):e2021049972. 34285080 10.1542/peds.2021-049972 PMC8344340

[20] Mackey K , Ayers CK , Kondo KK , et al. Racial and ethnic disparities in COVID-19–related infections, hospitalizations, and deaths: a systematic review. Ann Intern Med 2021;174:362–373. 33253040 10.7326/M20-6306 PMC7772883

[21] Van Dyke ME , Mendoza MCB , Li W , et al. Racial and ethnic disparities in COVID-19 incidence by age, sex, and period among persons aged <25 years—16 US jurisdictions, January 1–December 31, 2020. Morb Mortal Wkly Rep 2021;70:382–388. 10.15585/mmwr.mm7011e1 PMC7976617 33735165

[22] Eberly LA , Kallan MJ , Julien HM , et al. Patient characteristics associated with telemedicine access for primary and specialty ambulatory care during the COVID-19 pandemic. JAMA Netw Open 2020;3(12):e2031640. 33372974 10.1001/jamanetworkopen.2020.31640 PMC7772717

[23] Brown CL , Montez K , Amati JB , et al. Impact of COVID-19 on pediatric primary care visits at four academic institutions in the Carolinas. Int J Environ Res Public Health 2021;18:5734. 34071783 10.3390/ijerph18115734 PMC8199093

[24] Gerber JS , Prasad PA , Localio AR , et al. Racial differences in antibiotic prescribing by primary care pediatricians. Pediatrics 2013;131:677–684. 23509168 10.1542/peds.2012-2500 PMC9923585

[25] Fleming-Dutra KE , Shapiro DJ , Hicks LA , Gerber JS , Hersh AL. Race , otitis media, and antibiotic selection. Pediatrics 2014;134:1059–1066. 25404720 10.1542/peds.2014-1781

[26] Wattles BA , Vidwan NK , Feygin Y , Jawad KS , Creel LM , Smith MJ. Antibiotic prescribing to Kentucky Medicaid children, 2012–2017: prescribing is higher in rural areas. *J Rural Health* 2021. doi: 10.1111/jrh.12584. 10.1111/jrh.12584 33978987

[27] Milano B. With COVID spread, ‘racism—not race—is the risk factor.’ *The Harvard Gazette* website. https://news.harvard.edu/gazette/story/2021/04/with-covid-spread-racism-not-race-is-the-risk-factor/. Published April 22, 2021. Accessed February 22, 2022.

[28] Hersh AL , Stenehjem E , Daines W. RE: Antibiotic prescribing during pediatric direct-to-consumer telemedicine visits. Pediatrics 2019;144(2):e20191786B. 10.1542/peds.2019-1786B 31366684

[29] Ray KN , Shi Z , Gidengil CA , Poon SJ , Uscher-Pines L , Mehrotra A. Antibiotic prescribing during pediatric direct-to-consumer telemedicine visits. Pediatrics 2019;143(5):e20182491. 30962253 10.1542/peds.2018-2491 PMC6565339

[30] Ray KN , Martin JM , Wolfson D , et al. Antibiotic prescribing for acute respiratory tract infections during telemedicine visits within a pediatric primary care network. Acad Pediatr 2021;21:1239–1243. 33741531 10.1016/j.acap.2021.03.008

